# Optimizing the Colour and Fabric of Targets for the Control of the Tsetse Fly *Glossina fuscipes fuscipes*

**DOI:** 10.1371/journal.pntd.0001661

**Published:** 2012-05-29

**Authors:** Jenny M. Lindh, Parikshit Goswami, Richard S. Blackburn, Sarah E. J. Arnold, Glyn A. Vale, Mike J. Lehane, Steve J. Torr

**Affiliations:** 1 Vector Group, Liverpool School of Tropical Medicine, Liverpool, United Kingdom; 2 African Insect Science for Food and Health, Thomas Odhiambo Campus, Mbita Point, Kenya; 3 Sustainable Materials Research Group, Centre for Technical Textiles, University of Leeds, Leeds, United Kingdom; 4 Natural Resources Institute, University of Greenwich, Greenwich, United Kingdom; 5 Southern African Centre for Epidemiological Modelling and Analysis, University of Stellenbosch, Stellenbosch, South Africa; Johns Hopkins Bloomberg School of Public Health, United States of America

## Abstract

**Background:**

Most cases of human African trypanosomiasis (HAT) start with a bite from one of the subspecies of *Glossina fuscipes*. Tsetse use a range of olfactory and visual stimuli to locate their hosts and this response can be exploited to lure tsetse to insecticide-treated targets thereby reducing transmission. To provide a rational basis for cost-effective designs of target, we undertook studies to identify the optimal target colour.

**Methodology/Principal Findings:**

On the Chamaunga islands of Lake Victoria , Kenya, studies were made of the numbers of *G. fuscipes fuscipes* attracted to targets consisting of a panel (25 cm square) of various coloured fabrics flanked by a panel (also 25 cm square) of fine black netting. Both panels were covered with an electrocuting grid to catch tsetse as they contacted the target. The reflectances of the 37 different-coloured cloth panels utilised in the study were measured spectrophotometrically. Catch was positively correlated with percentage reflectance at the blue (460 nm) wavelength and negatively correlated with reflectance at UV (360 nm) and green (520 nm) wavelengths. The best target was subjectively blue, with percentage reflectances of 3%, 29%, and 20% at 360 nm, 460 nm and 520 nm respectively. The worst target was also, subjectively, blue, but with high reflectances at UV (35% reflectance at 360 nm) wavelengths as well as blue (36% reflectance at 460 nm); the best low UV-reflecting blue caught 3× more tsetse than the high UV-reflecting blue.

**Conclusions/Significance:**

Insecticide-treated targets to control *G. f. fuscipes* should be blue with low reflectance in both the UV and green bands of the spectrum. Targets that are subjectively blue will perform poorly if they also reflect UV strongly. The selection of fabrics for targets should be guided by spectral analysis of the cloth across both the spectrum visible to humans and the UV region.

## Author Summary

Efforts to control human African trypanosomiasis (HAT) would be strengthened by the development and application of more cost-effective methods of controlling the various species of tsetse fly vector. Among the most promising approaches is the use of insecticide-treated targets which use various olfactory and visual stimuli to attract and kill tsetse. Following on from previous studies of the responses of tsetse to odours and target size and shape, we compared the numbers of *G. f. fuscipes* attracted to different coloured targets. Our results show that the attraction of tsetse is correlated positively with reflectance in the blue region of the spectrum but negatively with the UV- and green regions. The best blue targets attract and kill three times more tsetse than the worst because of different UV reflectance levels in the different blue cloths. Hence selecting fabrics for use in targets must be based on spectral analysis of the fabrics' reflectance across the spectrum visible to tsetse, which includes UV, and not simply on the ‘rule of thumb’ that targets to control tsetse should be blue.

## Introduction

Tsetse flies (*Glossina* spp.) are restricted to sub-Saharan Africa where they transmit the trypanosomes causing the diseases of nagana in livestock and sleeping sickness, also known as human African trypanosomiasis (HAT), in humans. Tsetse are commonly divided into three groups: i) the Morsitans group (savannah species) which are the main vectors of the trypanosomes causing nagana; ii) the Palpalis group (riverine species) which are largely responsible for transmission of *Trypanosoma brucei* spp, the causative agents of HAT, and iii) the Fusca group (forest species) which currently are usually only minor vectors. There is no vaccine against trypanosomiasis, and the use of drugs is limited by problems of toxicity and resistance [1]. This, in addition to the fact that there are no prophylactic drugs available for humans, makes vector control particularly important. Given the distributions of tsetse vectors [2] and the incidence of HAT [3], [4], [5], it seems that at least 90% of all cases of HAT are transmitted by the subspecies of *Glossina fuscipes* (Palpalis group).

One of the most important methods of tsetse control is the use of stationary artificial baits, represented either by three-dimensional traps or, more cost-effectively, by two-dimensional cloth screens (targets) that are treated with insecticide [6]. Most of the work on the optimisation of target design has been performed with tsetse other than *G. fuscipes*, especially with the savannah species *G. morsitans morsitans* and *G. pallidipes* and the riverine species *G. palpalis palpalis* and *G. tachinoides* [7], [8], [9]. For these tsetse species the most effective target consists of black and/or phthalogen blue panels of cotton cloth, which traditionally have been used to make a target of about 1×1 m. The colour “phthalogen blue” produced by colouring processes based on pigment blue 15 (copper phthalocyanine) or its solubilized derivatives (turquoise blue) appears to be the optimal colour. This has been demonstrated in detailed comparisons of traps, fabrics, dyes and paints [10], [11]. Unfortunately, the most convenient locally-available blue fabric for tsetse applications, phthalogen blue cotton, has been difficult to obtain since the mid 1990's. Recently we have shown that for all subspecies of *G. fuscipes*, for *G. tachinoides* and *G. palpalis gambiensis*, but not for *G. m. morsitans* and *G. pallidipes* [12], the cost-effectiveness of a target can be improved several fold by using only phthalogen blue cloth, reducing its size by about 94%, to become 25×25 cm, and by adding a panel of fine black polyester netting of the same size [13], [14]. The distinctive optimum size of targets for Palpalis group flies suggests that targets for this group might also have a distinctive optimum colour. Moreover, even if blue panels were confirmed to be best for this group, it might be beneficial to opt for a fabric other than phthalogen blue cotton [10], [11] because of its limited availability. Polyester fabrics in particular have great potential as they are more suitable in most technical respects than cotton and can be produced cheaply. Under outdoor conditions polyester lasts about four times longer than cotton [15], which is particularly affected by UV degradation [15], mildew [16] and rot [17]. Colour fastness is easier to achieve in polyesters [18] and the amount of insecticide needed to impregnate polyester is less than for cotton [19]. Furthermore, polyester is easier to transport because it weighs less than cotton.

The present paper reports screening tests for the attractiveness of various colours and types of fabric in small cloth-and-net targets for *G. f. fuscipes*, one of the two most important vectors of sleeping sickness.

## Materials and Methods

### Targets and fabrics

Targets consisted of a 25×25 cm panel of cloth flanked by the same-sized panel of fine black polyester netting (Quality no. 166, Swisstulle, Nottingham, UK). Various materials, obtained from different sources, were used for the cloth panel. Phthalogen blue cotton (Phthalogen blue C) (used as the standard) and black cotton (Black 1) were already available at the research station and were from the same stocks used in previous studies of target design [13]. All other cotton materials (Brown, Orange, Red 1–2, Green 1–3, Yellow 1, Grey 1–2, Purple 1 and White 1) were bought in a textile shop in Sweden. The white cotton cloth (White 1) was washed with household bleach (Klorin^T^, Colgate-Palmolive, active ingredient: sodium hypochlorite) to create a material with high reflectance in the UV-region (supplementary Fig. S1 and S2). A black polyester (Black 2) and two blue polyester (Blue 7 and 8 called Phthalogen blue and Royal blue respectively by Vestergaard Frandsen Ltd, Lausanne (VF)) panels were made from materials identical to those used in the tsetse traps and targets produced by VF. The material called Phthalogen blue polyester by Vestergaard is dyed with a blue dye to create a polyester cloth of a colour similar to phthalogen blue cotton but it was not dyed with phthalogen blue dye which can only be used on cotton material. In addition, VF supplied polyester materials that were blue (Blue 2–6), purple (Purple 3–8), white (White 3) and yellow (Yellow 2). These polyester materials differed in weight, gloss and weave. Another seven polyester materials (Blue 9–13, Purple 2, White 2) were produced at the Centre for Technical Textiles, University of Leeds, by applying dyes (Appendix 1) to 100% polyester fabric (matt, texturized; knitted; 150 denier; 36 filaments; weight 114 g m^−2^) supplied by VF.

A total of 37 different materials were used. Their reflectance spectra were measured at the Danish technological service institute (http://www.dhigroup.com) on a Shimadzu dual beam photometer, from 190–900 nm at 10 nm intervals (supplementary Fig. S1).

### Catches and analyses

Studies were performed from February to December 2009 on Chamaunga Island (0.5 km^2^) (0° 25′S, 34°13′E), Lake Victoria, Kenya, using targets in which the cloth and netting panels were each covered on both sides with an electrocuting grid of fine black wires [20]. Tsetse knocked down by the grids fell into a tray of soapy water below each panel. In this way the catch from each panel could be recorded separately.

Fifteen separate experiments (supplementary Fig. S2) were conducted between 09.00 and 13.00 h, when *G. f. fuscipes* is most active [21], [22]. Each experiment involved five targets with different coloured cloth panels, which were compared in two blocks of Latin squares of 5 days ×5 sites, with sites at least 50 m apart. This produced a total of 10 daily replicates with each target. The sites were the same throughout the 15 experiments: none of the sites was shaded by vegetation and all targets were oriented the same way relative to the sun. All experiments were performed under dry conditions. The combined daily catch of the cloth and net panels (n) was transformed to log (n+1) for analysis of variance, the significance of differences between means being assessed by Tukey's Honest Significant Difference (HSD) test. All data analysis were performed using R [23]. Each experiment employed a target with Phthalogen blue C cloth as a standard, and the catches with the other targets were expressed as a proportion of the standard catch, to give a ‘catch index’. Thus a target that caught, say, twice as many tsetse as the standard would have a catch index of 2.0, and a target that caught only half that of the standard would have a catch index of 0.5.

Following earlier work [7], [8] we also assessed the effect of colour on landing response by comparing the proportion of the total catch taken from the coloured panel. The proportion is termed the ‘landing score’. The results were subjected to logistic regression with binomial errors using the statistical package R [23]. The catch of tsetse from (i) the target only and (ii) the target +flanking net were specified as the dependent variable and binomial denominator, respectively. Explanatory variables were the target colour, site and day. The significance of changes in deviance was assessed by either χ^2^ or, if the data were overdispersed, an *F*-test following re-scaling. The landing scores (reported in table 1 and 2) are accompanied by their sample size. For analyses of catch and landing, the term “significant” implies *P*<0.05.

**Table 1:** Catch index and landing score of different-coloured targets.

		Catch index		Landing score	
Exp	Colour	Males	Females	Males	Females
1	Standard	1.00 (14.4,1.19±0.108)	1.00 (12.5,1.13±0.084)	0.31 (157)	0.29 (137)
	Brown	0.84 (12.1) ns	0.84 (10.5) ns	0.29 (143) ns	0.29 (125) ns
	Red 2	0.75 (10.8) ns	0.67 (8.4) ns	0.33 (128) ns	0.29 (93) ns
	Red 1	0.44 (6.3)*	0.68 (8.5) ns	0.23 (75) ns	0.20 (90) ns
	Orange	0.72 (10.4) ns	0.55 (6.9) ns	0.19 (121) ns	0.21 (81) ns
2	Standard	1.00 (13.4,1.16±0.069)	1.00 (18.2,1.28±0.063)	0.32 (148)	0.36 (193)
	White 3	0.44 (5.9)***	0.30 (5.5)***	0.27 (67) ns	0.26 (61) ns
	Yellow 2	0.53 (7.1)**	0.32 (5.8)***	0.36 (76) ns	0.32 (65) ns
	Yellow 1	0.39 (5.2)***	0.32 (5.8)***	0.24 (59) ns	0.35 (63) ns
	Orange	0.46 (6.2)***	0.35 (6.4)***	0.40 (68) ns	0.44 (68) ns
3	Standard	1.00 (22.7,1.37±0.071)	1.00 (27.0,1.45±0.095)	0.27 (240)	0.22 (281)
	Green 1	0.43 (9.8)***	0.38 (10.3)**	0.12 (106) *	0.11 (108) ns
	Green 2	0.67 (15.2) ns	0.41 (11.1)**	0.08 (165) **	0.08 (158) **
	Green 3	0.48 (10.9)**	0.42 (11.3)**	0.08 (121) *	0.10 (149) *
	Purple 1	0.85 (19.3) ns	0.47 (12.7)*	0.16 (210) *	0.11 (176) *
4	Standard	1.00 (16.6,1.25±0.089)	1.00 (27.0,1.45±0.061)	0.21 (184)	0.22 (289)
	Blue 10	0.49 (8.1)*	0.51 (13.8)***	0.15 (96) ns	0.13 (147) ns
	Blue 12	0.69 (11.5) ns	0.48 (13.0)***	0.16 (135) ns	0.15 (144) ns
	Green 2	0.54 (9.0) ns	0.55 (14.9)**	0.20 (118) ns	0.19 (162) ns
	Orange	0.52 (8.6)*	0.42 (11.3)***	0.18 (96) ns	0.14 (125) ns
5	Standard	1.00 (14.2,1.18±0.084)	1.00 (22.8,1.38±0.064)	0.27 (177)	0.20 (259)
	Brown	0.75 (10.7) ns	0.54 (12.3)**	0.25 (146) ns	0.32 (136) ns
	Red1	0.55 (7.8) ns	0.42 (9.6)***	0.24 (92) ns	0.18 (122) ns
	Black 1	0.65 (9.2) ns	0.35 (8.0)***	0.21 (112) ns	0.28 (94) ns
	Purple 1	0.82 (11.6) ns	0.43 (9.8)***	0.24 (142) ns	0.21 (113) ns
6	Standard	1.00 (20.7,1.34±0.052)	1.00 (17.9,1.28±0.055)	0.34 (228)	0.32 (189)
	Black 1	0.55 (11.4)***	0.57 (10.2)**	0.32 (116) ns	0.32 (108) ns
	Grey 1	0.30 (6.2)***	0.35 (6.3)***	0.25 (63) ns	0.27 (64) ns
	Grey 2	0.36 (7.5)***	0.38 (6.8)***	0.24 (78) ns	0.26 (72) ns
	White 2	0.32 (6.6)***	0.31 (5.5)***	0.28 (71) ns	0.21 (62) ns
7	Standard	1.00 (10.3,1.05±0.088)	1.00 (14.7,1.20±0.090)	0.32 (123)	0.28 (165)
	White 2	0.58 (6.0) ns	0.71 (10.4) ns	0.08 (83) ns	0.07 (138)**
	White 1	0.82 (8.4) ns	0.45 (6.6)*	0.23 (106) ns	0.32 (82) ns
	Grey 1	0.50 (5.2)*	0.45 (6.6)*	0.24 (59) ns	0.23 (73) ns
	Black 2	0.85 (8.8) ns	0.85 ns (12.5)	0.21 (104) ns	0.25 (138) ns

Catch index is the detransformed mean daily catch of a target expressed as a proportion of that from the standard target (Phthalogen blue cotton). The detransformed mean catches are shown in brackets. For the Standards only, the transformed mean catch±SED are also reported. The landing score (sample size in brackets) of each cloth is the number landing on the target expressed as a proportion of the total number caught by the bait. Asterisks indicate that the catch index or landing score differs from the standard at 0.05 (*), 0.01 (**) or 0.001(***) levels of probability.

**Table 2:** Catch and landing score of tsetse from various blue- and purple-coloured targets.

		Catch index		Landing score	
Exp.	Colour	Males	Females	Males	Females
8	Standard	1.00 (20.0, 1.32±0.062)	1.00 (19.8, 1.32±0.061)	0.39 (216)	0.33 (208)
	Black 1	0.49 (9.8)***	0.47 (9.9)***	0.30 (105) ns	0.35 (105) ns
	Blue 7	0.51(10.2) ***	0.42 (8.3)***	0.17 (112) **	0.19 (90) *
	Blue 8	0.48 (9.6)***	0.49 (9.7)***	0.12 (104) ***	0.11 (104) **
	Black 2	0.40 (8.0)***	0.35 (6.9)***	0.29 (85) ns	0.24 (79) ns
9	Standard	1.00 (11.6, 1.10±0.061)	1.00 (15.3, 1.21±0.067)	0.45 (130)	0.37 (166)
	Blue 7	0.68 (7.9) ns	0.60 (9.2)*	0.28 (85) *	0.32 (94) ns
	Blue 9	0.62 (7.2)*	0.58 (8.9)*	0.33 (76) ns	0.21 (94) *
	Blue 12	0.66 (7.7) ns	0.61 (9.3)*	0.26 (85) **	0.27 (100) ns
	Blue 13	0.56 (6.5)**	0.57 (8.7)*	0.29 (72)*	0.25 (101) *
10	Standard	1.00 (15.3, 1.21±0.061)	1.00 (19.8, 1.32±0.073)	0.36 (157)	0.25 (205)
	Blue 8	0.62 (9.5)*	0.51 (10.1)**	0.19 (112) ns	0.23 (105) ns
	Blue 2	0.47 (7.2)***	0.38 (7.5)***	0.27 (82) ns	0.39 (82) ns
	Blue 3	0.47 (7.2)***	0.46 (9.1)**	0.29 (76) ns	0.23 (99) ns
	Blue 4	0.41 (6.3)***	0.34 (6.7)***	0.30 (70) ns	0.23 (73) ns
11	Standard	1.00 (20.7, 1.34±0.079)	1.00 (29.7, 1.49±0.063)	0.35 (215)	0.25 (308)
	Blue 4	0.39 (8.1)***	0.41 (12.2)***	0.27 (86) ns	0.28 (137) ns
	Blue 5	0.31 (6.4)***	0.29 (8.6)***	0.20 (70) ns	0.34 (89) ns
	Purple 2	0.69 (14.3) ns	0.55 (16.3)**	0.30 (162) ns	0.16 (188) ns
	Blue 9	0.36 (7.5)***	0.36 (10.7)***	0.26 (82) ns	0.25 (112) ns
12	Standard	1.00 (24.7, 1.41±0.076)	1.00 (21.3, 1.35±0.065)	0.38 (253)	0.43 (204)
	Blue 11	0.23 (5.7)***	0.35 (7.5)***	0.19 (68) **	0.18 (71) ***
	Blue 10	0.25 (6.2)***	0.37 (7.9)***	0.21 (78) *	0.28 (83) *
	Blue 6	0.44 (10.9)**	0.32 (6.8)***	0.19 (119) **	0.28 (75) *
	Purple 2	0.80 (19.8) ns	0.77 ns (16.4)	0.36 (204) ns	0.35 (159) ns
13	Standard	1.00 (19.4, 1.31±0.056)	1.00 (25.3, 1.42±0.063)	0.37 (206)	0.34 (258)
	Purple 1	0.51 (9.9)***	0.45 (11.4)***	0.29 (108) ns	0.30 (119) ns
	Purple 2	0.63 (12.2)*	0.67 (17.0) ns	0.31 (140) ns	0.28 (191) ns
	Blue 7 1^a^	0.48 (9.3)***	0.42 (10.6)***	0.31 (98) ns	0.29 (107) ns
	Blue 7 3^a^	0.34 (6.6)***	0.35 (8.9)***	0.31 (71) ns	0.21 (90) ns
14	Standard	1.00 (14.3, 1.18±0.070)	1.00 (22.4, 1.37±0.068)	0.28 (168)	0.19 (263)
	Purple 4	0.73 (10.4) ns	0.62 (13.9) ns	0.26 (124) ns	0.15 (161) ns
	Purple 5	0.81 (11.6) ns	0.52 (11.6)**	0.20 (131) ns	0.18 (131) ns
	Purple 8	0.74 (10.6) ns	0.59 (13.2)*	0.22 (117) ns	0.15 (142) ns
	Purple 2	0.73 (10.4) ns	0.58 (13.0)*	0.24 (127) ns	0.18 (139) ns
15	Standard	1.00 (22.1, 1.36±0.066)	1.00 (28.9, 1.48±0.075)	0.27 (233)	0.25 (318)
	Purple 3	0.54 (11.9)**	0.50 (14.5)**	0.24 (136) ns	0.20 (175) ns
	Purple 6	0.44 (9.7)***	0.46 (13.3)**	0.14 (106) ns	0.13 (159) ns
	Purple 7	0.50 (11.1)**	0.52 (15.0)**	0.21 (146) ns	0.13 (196) ns
	Purple 1	0.56 (12.4)**	0.48 (13.9)**	0.15 (157) ns	0.17 (165) ns

a 1 refers to 1 layer of cloth, 3 refers to 3 layers of cloth.

Catch index is the detransformed mean daily catch of a target expressed as a proportion of that from the standard target (Phthalogen blue cotton). The detransformed mean catches are shown in brackets. For the Standards only, the transformed mean catch±SED are also reported. The landing score (sample size in brackets) of each cloth is the number landing on the target expressed as a proportion of the total number caught by the bait. Asterisks indicate that the catch index or landing score differs from the standard at 0.05 (*), 0.01 (**) or 0.001(***) levels of probability.

### Modelling target catch as a function of spectral reflectivities

Multiple regression analyses were done in R to examine the relationship between the catch index and mean reflectance of all the 37 materials utilised in this study, in four colour bands which broadly matched those used in previous studies: 300–400 nm (‘ultraviolet’), 410–500 nm (‘blue’), 510–600 nm (‘green’) and 610–700 nm (‘red’) [7], [24]. In addition, multiple regression analyses with percentage reflectance at four wavelengths (330, 355, 460 and 520 nm) as explanatory variables were performed. Tsetse flies, like most higher flies, are believed to possess four photoreceptor types in their eyes. These four wavelengths were selected as being representative of the peak sensitivities of the four photoreceptor types, as indicated by previous studies [25], [26]. Therefore they provide a measure of the stimulation of each photoreceptor by the 37 different fabric panels, which can be evaluated individually and relative to the other receptors, as in fly colour models [27]. Following earlier work [24], logs were taken of both the target catch index and percent reflectivity as the relationship was found to be log-linear. Explanatory variables were removed from a model in which all terms were fitted without any interactions. Terms that reduced deviance significantly from the model were then used in a maximal model in which all terms were fitted with all their interactions. Non-significant interaction terms were removed by a series of *F*- tests commencing with terms having the highest order of interaction and least significance. Only terms that reduced deviance significantly from the maximal model were included in the final, minimally-adequate model.

## Results

### Comparison of different colours

**Figure 1:**
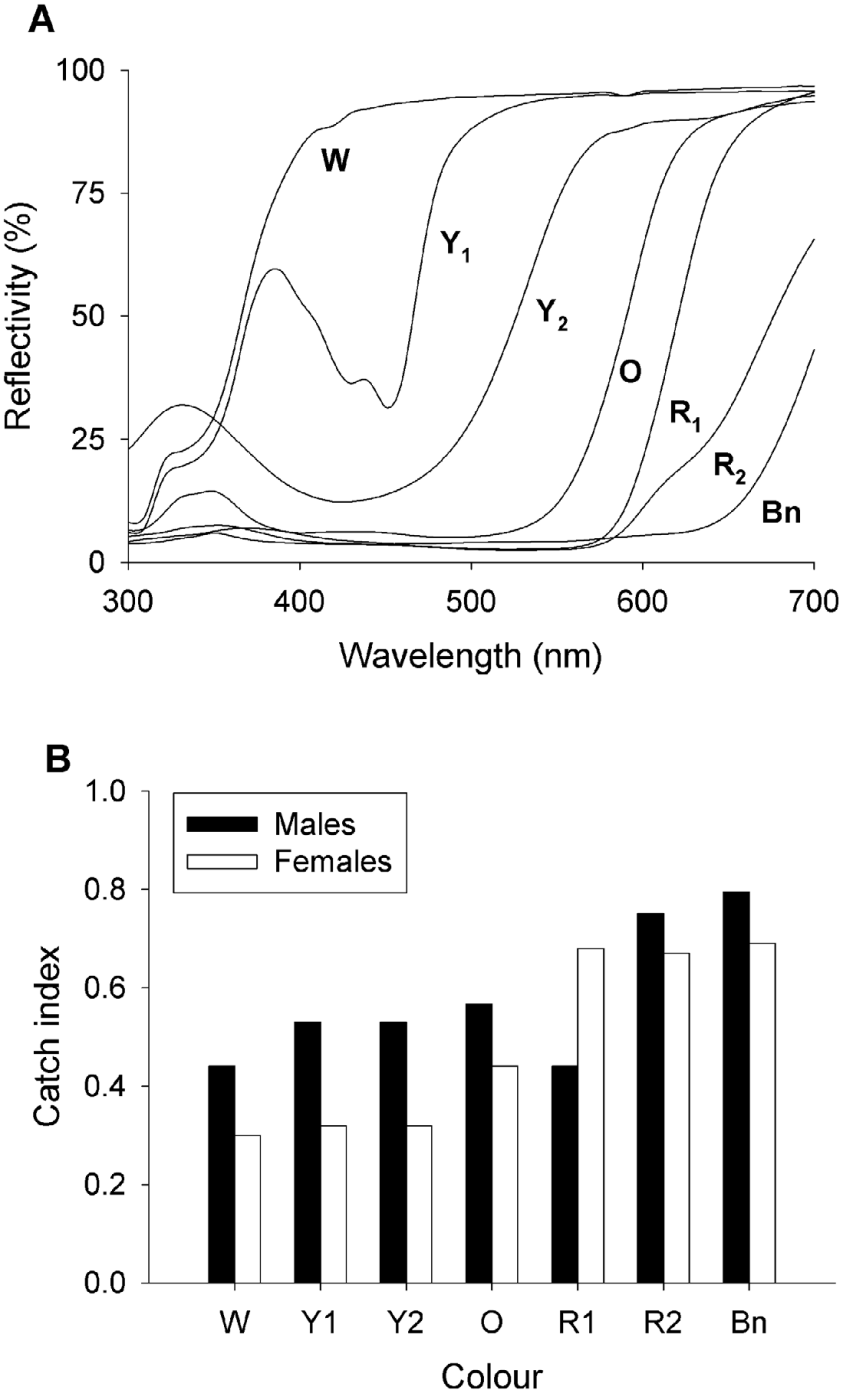
Spectral reflectivity and mean catches of tsetse from ‘cut-off’ coloured targets. (A) Spectral reflectivity of White 3 (W), Yellow 1 and 2 (Y_1_, Y_2_), Orange (O), Red 1 and 2 (R_1_, R_2_) and Brown (Bn). (B) Mean catches of male and female tsetse based on catch indices presented in Table 1.

**Figure 2:**
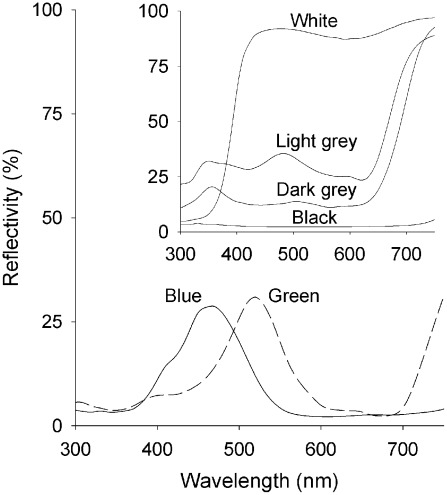
Spectral reflectivities of ‘band-pass’ and achromatic targets. Main figure shows spectra for the standard (Phthalogen blue) and green (Green 2) targets. Inset shows Black 1, White 2 and Grey 1 and 2 (G1, G2) targets.

The first set of seven experiments (Table 1) compared the responses of *G. f. fuscipes* to baits of different colour. The colours can be divided into two groups: i) “cut-off” colours, i.e., colours with a steeply sloped spectrum (yellow, orange, red and brown, Fig. 1 and Supplementary Fig. S1), and ii) “band reflecting” colours (i.e. blue, green Fig. 2 and Supplementary Fig. S1 and S2, [24]). The total catches suggest that no colour was significantly better than the Phthalogen blue C standard (Table 1) – the index with other colours being on average only 0.58 (range: 0.30–0.85) for males and 0.48 (range: 0.30–0.85) for females, albeit that the index was not always significantly different from the Phthalogen blue C standard of 1.00. “Cut off” colours, with spectra of slope >500 nm, had efficacies that more closely approached the Phthalogen blue C standard, being on average 0.63 (range: 0.44–0.84) for males and 0.56 (range: 0.35–0.84) for females (Exp. 1, 2, 4 and 5, Fig. 1B). Yellow and green targets performed poorly, with an average index of 0.51 (range: 0.39–0.67) for males and 0.40 (range: 0.32–0.55) for females (Exp. 2–4), while purple (Purple 1) was highly effective for male (average 0.84, range: 0.82–0.85) flies but not females (average 0.45, range: 0.43–0.47, Exp. 3 and 5).

The series of achromatic targets (see Fig. 2 inset) compared in two experiments indicated that effectiveness tended to decline in the order black, dark grey, light grey and white (Exp. 6 and 7), albeit that the index for White 2 differed almost two-fold between experiments. Since the catch with the Phthalogen blue C standard was higher than with any of the achromatic colours it seems that the effectiveness of the blue is dependent on colour discrimination rather than intensity contrast alone.

In general the proportion caught on the cloth (the landing score) was low for all colours (Table 1), averaging 0.24 (range: 0.08–0.40) for males and 0.23 (range: 0.07–0.44) for females. The lowest proportions being observed for three shades of green cotton (Green 1–3) and a purple cotton (Purple 1) material in Exp. 3 and a white polyester (White 2) in Exp. 7 (Table 1). These were the only experiments where a significant difference in landing score compared to the standard was observed. However, the landing score for three of the same materials was not significantly different to the standard in three other experiments (comp. Exp. 4 for Green 2, Exp. 5 for Purple 1 and Exp. 6 for White 2.

### Comparison of blue and purple fabrics

The second set of experiments (Table 2) focused on the blue, purple and black colours that performed well in the first set (Table 1), but explored a wider range of materials. Again the Phthalogen blue C standard performed better than any other cloth. A higher peak at the reflectance accounting for most of the reflectance of Phthalogen blue C standard (supplementary Fig. S2) did not increase the catch (Exp. 9). The same was observed in experiment 10 which compared blue materials with reflectance peaks at a slightly lower wavelength than the Phthalogen blue C standard (among them Blue 8). This contrasts with a previous study [24] which found a positive linear relationship between log transformed blue reflectance of the materials used for traps and the log transformed catches of the traps. This difference may be explained by the higher reflectance in the UV range for all the materials compared to the standard and Blue 8 respectively (Supplementary Fig. 2). The Phthalogen blue C standard was about twice as effective as the corresponding polyester cloth (Exp. 8, 9 and 13). This poor performance of polyester did not seem to be due to the relatively high translucence of the fabric since reducing the translucence of the polyester, by using three layers together, did not increase the catch, in fact it lowered it. This decrease in fly numbers was significant for males (Exp. 13).

Of all the fabrics tested the most promising alternative to the Phthalogen blue C standard was purple polyester (Purple 2, Exp. 11, 12 and 14). Furthermore, the purple polyester bait performed well in relation to the blue polyester material (Blue 7). Two experiments compared different shades of purple polyester cloth (Exp. 14 and 15). For males, Purples 4, 5 and 8 had high catch indices (Table 2), while Purples 3, 6 and 7 performed less well. The latter three purples were comparatively dark, with reflectance peaking at relatively lower wavelengths (Supplementary Fig. S2). For females the indices seemed little affected by the type of purple.

As with the first set of experiments (Table 1), the second set (Table 2) showed that a relatively low proportion of tsetse landed on the cloth panel. Overall the landing score was highest with the standard (range: 0.28–0.45 for males, 0.19–0.43 for females). The significantly lower landing score observed for some materials (Exp. 8, 9 and 12) was mainly for blue polyester cloth which had a reflectance peak at the same wavelength as the standard but with a higher peak (Blue 7, 9, 10, 11, 12 and 13, Supplementary Fig. S2). However, as in the first set of experiments the significantly lower landing response was not consistent between experiment (comp. Blue 7 in Exp. 8 and 9 to Exp. 13).

### Modelling target catch as a function of spectral reflectivities

Details of the regression models are shown in Table 3. Modelling catch as a function of reflectance in the various colour bands showed that for both sexes, catch was negatively correlated with reflectivity in the ‘ultraviolet’ and ‘green’ bands but positively correlated with reflectivity in the ‘blue’ band. For females only, there was also a positive correlation with reflectivity in the ‘red’ band. Carrying out the regression analysis with reflectance at four wavelengths where tsetse show peak sensitivities showed that reflectivity at 360 nm, 460 nm and 520 nm were highly significant and exhibited the same trend as the analyses with colour bands: catches were negatively correlated with reflectivity at 360 nm (≈UV) and 520 nm (≈green) but positively correlated with 460 nm (≈blue). The ‘band’ (regressions 1 and 2) and ‘peak’ models (regressions 3 and 4) explained similar amounts of variation (40–42% for the male catches and 61–62% for females). For both the ‘colour band’ and ‘wavelength’ models, there were no significant interactions between the main explanatory variables.

**Table 3:** Regression analyses of relationship between catch and spectral reflectivities of targets.

	Sex	Model	Deviance	Δ deviance (%)	d.f.	*F* to remove	Estimate	SE
**1**	Males	Null	1.955		74			
								
		Maximal	1.140		70			
		-UV	0.294	25.8	1	18.1***	−0.323	0.0738
		-Blue	0.230	20.2	1	14.1***	0.185	0.0495
		-Green	0.086	7.5	1	5.3*	−0.108	0.0589
		-Red	0.034	3.0	1	2.1 ns		
		(Intercept)					−0.015	0.0589
**2**	Females	Null	2.060					
		Maximal	0.776					
		-UV	0.485	62.5	1	43.8***	−0.536	0.0810
		-Blue	0.384	49.5	1	34.6***	0.322	0.0547
		-Green	0.119	15.3	1	10.8**	−0.180	0.0548
		-Red	0.052	6.7	1	4.6*	0.088	0.0407
		(Intercept)					−0.038	0.0465
**3**	Males	Null	1.955		74			
		Maximal	1.061		70			
		−λ330	0.060	5.7	1	3.9 ns		
		−λ360	0.177	16.7	1	11.7**	−0.261	0.0463
		−λ460	0.232	21.9	1	15.3***	0.202	0.0612
		−λ520	0.152	14.3	1	10.0**	−0.171	0.0682
		(Intercept)					−0.026	0.0540
**4**	Females	Null	2.060		74			
		Maximal	0.728		70			
		−λ330	0.037	5.1	1	3.5 ns		
		−λ360	0.178	24.5	1	17.1***	−0.261	0.0463
		−λ460	0.297	40.8	1	28.5***	0.202	0.0612
		−λ520	0.162	22.3	1	15.6**	−0.171	0.0681
		(Intercept)					−0.026	0.0540

For each regression model, explanatory terms (mean reflectivities across colour bands for regressions 1 and 2 or at particular wavelengths for regressions 3 and 4) were removed from the maximal models in which all terms were included but not their interactions. Parameter estimates and SEs are for the minimally-adequate models which include significant terms only. The change in deviance (**Δ deviance)** due to removing a term from the maximal model is also expressed as a percentage of the deviance of the maximal model.

## Discussion

The results show that the responses of *G. f. fuscipes* to colour are broadly similar to those of other tsetse: blues, and phthalogen blue *sensu stricto* in particular, are more attractive than other colours whereas reds and blacks are intermediate and green-yellow is least attractive [7], [8]. In studies of *G. pallidipes* in Zimbabwe, [24] catch was modelled from different coloured traps as a function of mean reflectivity in four colour bands: 300–410 nm (ultraviolet), 410–520 nm (blue-green), 520–615 nm (green-yellow-orange) and 615–700 nm (red). A similar approach was followed with studies of *G. palpalis palpalis* in Côte d'Ivoire using three colour bands: 300–380 nm (ultraviolet), 380–480 nm (ultraviolet-blue) and 480–620 nm (blue-green-yellow- red) [7]. Furthermore, physiological studies of the eyes of tsetse [26], [28] and other higher Diptera such as *Musca* [25] suggest that they have four peaks of sensitivity at 330 nm, 360 nm, 460 nm and 520 nm. Consequently these four bands and four reflectivity peaks were used in multiple regression analysis in this study. The results of these analyses, which show a negative correlation with ‘ultraviolet’ (band and peak), green (peak) and ‘green’ (band) reflectivity and a positive correlation with ‘blue’ (band and peak) reflectivity, are in accordance with those for *G. pallidipes* [24] and *G. palpalis palpalis* [7].

Even though our data show that there are many similarities between the response of *G. f. fusipes* and other tsetse species to visual cues, the data also confirms the observed differences between Palpalis and Morsitans group tsetse flies. In the present study there is a 2–3× difference in catch between the best (phthalogen blue cotton) and worst (yellow and targets with high UV reflectance) targets. This range is similar to that reported for other Palpalis-group tsetse [7], [8] but much less than the ten-fold range reported for Morsitans-group tsetse [29]. It seems likely therefore that the Palpalis-group tsetse are less responsive than the Morsitans-group to colour. Furthermore, Morsitans-group tsetse are equally attracted to black and blue targets, and black elicits a stronger landing response [29], which contrasts with the landing scores reported here. Previous studies show that for Palpalis group tsetse, (*G. p. palpalis and G. tachinoides*) phthalogen blue is more attractive than black, and black does not seem to elicit a marked landing responses [7], [8]. Our data confirm these results for *G. f. fuscipes* (Exp. 5–7, Table 1 and Exp. 8, Table 2).

The landing score was in general low in this study and it did not increase with the greater UV reflectance of white (Exp. 7 Table 2), as was observed for *G. p. palpalis, G. tachinoides* and *G. pallidipes* in previous studies [7], [8], [30], [31].

The widespread attraction of tsetse, along with many other species of biting Diptera, to blue and black objects is intriguing. It has been suggested that the contrast of blue against the green-yellow reflectance of vegetation is essentially a stimulus of ‘not vegetation’ [32]. More recently, it has been suggested that this phenomenon is related to the resting behaviour of tsetse; tsetse commonly rest in shady places which are tinted bluish by the scattered blue skylight [33]. However, tsetse attracted to targets are generally in a host- and/or mate-seeking mode of behaviour rather than seeking a resting site [8], [34] and thus it seems unlikely that tsetse are ‘mistaking’ targets for shady places. Nonetheless, hosts themselves are characterised by shaded areas, particularly those on the underside of their bodies – hence the suggestion that countershading has evolved to conceal prey from predators [35]. The response to blue may therefore be related, at least in part, to the shadows created on the bodies of potential hosts.

The data presented support the view that phthalogen blue cotton is at least as effective as any other material tested, and probably more effective than most or all of them. This is unfortunate given the declining availability of phthalogen blue dye, the technical problems with cotton, and the difficulties of dyeing artificial fibres with phthalogen blue. There seem to be two main options. First, it might be useful to look for other alternatives to phthalogen blue dyes – ones that can be used with polyester. Scientists have searched for such options previously [10], [11]. Present experiments indicate the wavelengths on which such a search should concentrate for *G. fuscipes*. The purple-blue range (370–470 nm) and red range (>500 nm, for “cut off” colours) was much more effective for males and females than the yellow-green range (525–600 nm, for “band reflecting” colours). Furthermore, light blue fabrics (Blue 4, Blue 5 and Blue 11) were in general of poor effectiveness. Our results underline the important negative effects of UV reflectivity on the attraction of tsetse to targets. Hence the selection of fabrics must be guided by spectral analysis and not just visual inspection of the cloth to identify fabrics that reflect strongly in the blue- but weakly in the UV-region of the spectrum. Second, if the only highly effective and colour-fast dye that is available can be used only on cotton, it might be acceptable to employ targets where the cloth panel is not treated with insecticide. Previous work has shown that treating a net with 0.8% deltamethrin results in >70% mortality for at least 9 months [36]. Present data for the distribution of catches between the netting and cloth panels (the landing score) suggest that the loss of effectiveness due to not treating the cloth will not be greater than about a third, and the loss might be much less if, as expected [37], many or most of the flies that alight first on the cloth panel subsequently fly round it and so collide with the net before departing from the target site.

In any event, allowing that it might be useful to screen cloth materials for use with those types of target in which only the netting is impregnated with insecticide, it would be useful if future screening tests employed not only the present fully-electrified targets but also targets in which the grid is restricted only to the net.

## Supporting Information

Appendix S1 **Dying procedure of polyester materials Blue 9–13, Purple 2, White 2.** Detailed dying procedure for materials Blue 9–13, Purple 2, White 2.
(DOCX)

Figure S1 **Reflectance spectra data.** Data for reflectance spectra of the 37 different materials used measured at Danish technological service institute (http://www.dhigroup.com) on a Shimadzu dual beam photometer, from 190–900 nm at 10 nm intervals.
(XLSX)

Figure S2 **Reflectance spectra graphs for each experiment.** Graphs of spectral reflectivities of materials used in each experiment, on the y-axis is percent reflectivity and on the x-axis wavelength in nm.
(XLSX)
