# Supplementary material for: Optimizing the Colour and Fabric of Targets for the Control of the Tsetse Fly Glossina fuscipes fuscipes
Source: PLoS Negl Trop Dis. 2012 May 29;6(5):e1661. doi: 10.1371/journal.pntd.0001661 (PMC3362611; doi:10.1371/journal.pntd.0001661)
Supplement: Appendix S1 — Dying procedure of polyester materials Blue 9–13, Purple 2, White 2. Detailed dying procedure for materials Blue 9–13, Purple 2, White 2. (DOCX) [file pntd.0001661.s001.docx]

**APPENDIX S1**

Prior to dyeing, polyester fabric samples (5 g) were scoured using 2 g dm^-3^ sodium carbonate and 1 g dm^-3^ *Kieralon MFB* (BASF) using a 20:1 liquor ratio at 60 °C for 15 min. Samples were scoured in stainless steel, sealed dye tubes in a laboratory-scale *Roaches Pyrotec 2000* dyeing machine. At the end of the treatment, the samples were rinsed with water and dried in air. Scoured fabric samples (5 g) were dyed at a concentration of 2% on mass of fibre (omf) using a liquor: fibre ratio (LR) of 20:1. The disperse dyes *Teratop Violet BL* (Purple 2; C. I. Disperse Violet 57; anthraquinone derivative), *Teratop Blue BGE* (Blue 9; C.I. Disperse Blue 60; **1**), *Teratop Blue HL-GR* (Blue 10; no C.I. name available), *Teratop Blue GLF* (Blue 11; C.I. Disperse Blue 27; **2**), *Teratop Blue B2F* (Blue 12; no C.I. name available), and *Teratop Blue HL-B* (Blue 13; C.I. Disperse Blue 54; anthraquinone derivative), and the UV-absorber *UV Fast P* (White 2), were all supplied by Huntsman Textile Effects. These specific dyes were chosen because of their high light fastness, which is desirable for this specific application. Dyeing assistants added were 1 g dm^-3^ *Levagal DLP* (Lanxess) and 2 g dm^-3^ *Ludigol AR* (BASF). The pH was adjusted to 4.5-5.0 using 0.2 M Na_2_HPO_4_ and 0.1 M citric acid (McIlvaine) buffer. Samples were dyed in stainless steel, sealed dye tubes in a laboratory-scale *Roaches Pyrotec 2000* dyeing machine. The dyebath was heated steadily (2 °C min^-1^) until the dyeing temperature was achieved (130 °C), then held at that temperature for 45 min. After dyeing, the dyebaths were cooled; dyed samples were rinsed with water and left to dry in air under ambient conditions. Reduction clearing was conducted to remove any dye that had not diffused into the fibres and was deposited on the surface. Dyed fabric samples were treated with 1.5 g dm^-3^ sodium carbonate and 2.0 g dm^-3^ sodium hydrosulfite using a LR of 20:1 in stainless steel, sealed dye tubes in a laboratory-scale *Roaches Pyrotec 2000* dyeing machine. The dyebath was heated rapidly to 60 °C then held at that temperature for 15 min. After treatment, the samples were rinsed with water and left to dry in air under ambient conditions.
